# Discrepancies between CSF biomarker and PET determinations of elevated brain amyloid and their prognostic significance

**DOI:** 10.1002/alz.70468

**Published:** 2025-07-09

**Authors:** David S. Knopman, Stephen D. Weigand, Heather J. Wiste, Jonathan Graff‐Radford, Neill R. Graff‐Radford, Ronald C. Petersen, Bradley F. Boeve, Clifford R Jack Jr, Val J. Lowe, Mary M. Machulda, Julie A. Fields, Vijay K. Ramanan, Hugo Botha, Stuart J. McCarter, David T. Jones, Bryan J. Neth, Gregory S. Day, Kejal Kantarci, Alicia Algeciras‐Schimnich, Joshua A. Bornhorst, Derek R. Johnson, and the Alzheimer's Disease Neuroimaging Initiative

**Affiliations:** ^1^ Department of Neurology Mayo Clinic Rochester Minnesota USA; ^2^ Department of Quantitative Health Sciences Mayo Clinic Rochester Minnesota USA; ^3^ Department of Neurology Mayo Clinic Jacksonville Florida USA; ^4^ Department of Radiology Mayo Clinic Rochester Minnesota USA; ^5^ Department of Psychiatry and Psychology Mayo Clinic Rochester Minnesota USA; ^6^ Department of Laboratory Medicine and Pathology Mayo Clinic Rochester Minnesota USA

**Keywords:** Alzheimer's disease, amyloid PET imaging, CSF amyloid biomarkers, longitudinal outcomes, mild cognitive impairment

## Abstract

**INTRODUCTION:**

When cerebrospinal fluid (CSF) and positron emission tomography (PET) measurements for amyloid‐beta‐peptide (Aβ) related pathology are discordant, therapeutic decision‐making becomes uncertain.

**METHODS:**

Using data from patients with mild cognitive impairment (*n* = 541) from the Alzheimer's Disease Neuroimaging Initiative, we examined baseline characteristics and longitudinal clinical outcomes in persons grouped according to normal/abnormal Aβ via concurrent CSF and PET determinations using standard cutpoints.

**RESULTS:**

Discordant groups for brain Aβ status (CSF+/PET− and CSF−/PET+) each represented about 5% of the mild cognitive impairment (MCI) population. Longitudinally, neither discordant group declined more than the CSF−/PET− group on either a memory measure or the Clinical Dementia Rating Sum of Boxes scores over a median 4 years of observation, while the CSF+/PET+ group exhibited worsening on both measures.

**DISCUSSION:**

In contrast to the clinical decline observed in the CSF+/PET+ group, persons with MCI and CSF+/PET− or CSF−/PET+ brain amyloid patterns did not exhibit incipient decline.

**Highlights:**

- Discrepant abnormal cerebrospinal fluid (CSF) and positron emission tomography (PET) brain amyloid indicators are uncommon in mild cognitive impairment (MCI).
- CSF‐PET discrepant persons with MCI tend to have less abnormal values initially.
- CSF‐PET discrepant persons with MCI have a benign prognosis at 4 years.

## BACKGROUND

While cerebrospinal fluid (CSF) assays (Aβ42/40 or p‐tau181/Aβ42 ratios) to detect elevated brain amyloid‐beta‐peptide (Aβ) are highly concordant with amyloid positron emission tomography (PET) ‐based measurements,^1^, ^2^, ^3^, ^4^, ^5^, ^6^, ^7^ there is a small fraction of persons in whom CSF and PET findings are discordant. In the era of anti‐amyloid monoclonal antibody (AAMA) therapeutics, where either abnormal CSF Aβ determinations or elevated amyloid PET determinations are considered equivalent for qualifying a prospective patient for treatment with an AAMA, discordant CSF and PET measurements for elevated brain Aβ may present a conundrum for therapeutic decision‐making. Information about subsequent cognitive decline for different CSF/PET abnormality patterns would resolve the question, but we are unaware of studies that reported outcomes according to jointly considered CSF and PET determinations of brain Aβ status. We focused on persons with mild levels of cognitive impairment (MCI) because such individuals are the ones who would currently be eligible for therapy contingent on elevated brain Aβ. We examined longitudinal outcomes from the Alzheimer's Disease Neuroimaging Initiative (ADNI) study in MCI patients with a particular focus on comparing persons with discrepant CSF Aβ and Aβ PET to those with concordant CSF and PET brain amyloid measures.

## 1 METHODS

### 1.1 Participants

We used ADNI data downloaded from LONI (http://adni.loni.usc.edu/) on April 30, 2024, from ADNIGO, ADNI2, and ADNI3, and considered all persons who received clinical diagnoses of MCI. Clinical diagnoses were performed using previously published procedures in ADNI.^8^ To be included, participants had to have a baseline neuropsychological battery, a CSF examination, a baseline amyloid PET scan, and at least one follow‐up visit that included the neuropsychological battery and a clinical diagnosis.

We did not include persons with mild dementia because of the rarity of discordant cases in the ADNI mild dementia category (no persons with the CSF−/PET+ pattern and only 10 with the CSF+/PET− pattern).

### 1.2 Instruments

The sum of trials 1–5 from the Auditory Verbal Learning test (AVLT) and a Clinical Dementia Rating Sum of Boxes scores (CDRsb)^9^ were selected as outcome measures. The AVLT sum of trials was selected because it is a core memory impairment marker with a dynamic nature in MCI and because of its psychometric properties including good test–retest reliability.^10^ The AVLT sum of trials 1‐5 has a range of 0–75 words.

### 1.3 Imaging and spinal fluid

Amyloid PET scans were performed with either florbetapir (89%) or florbetaben (11%) tracers. We defined an *abnormal amyloid PET (PET+)* as Centiloid (CL) ≥ 25 based on a global/meta‐region of interest (ROI) determined from the amyloid PET scan (processed at University of California [UC], Berkeley).^11^, ^12^ The ADNI FreeSurfer 5.3 pipeline yields a global standardized uptake value ratio (SUVR) measure representing a non‐weighted average of radiotracer retention in four FreeSurfer‐defined regions (frontal, anterior/posterior cingulate, lateral parietal, and lateral temporal cortices) normalized to whole cerebellum as previously described.^13^ The CL values were from harmonized 6 mm smoothed results provided by UC Berkeley.

The Roche Elecsys electrochemiluminescence immunoassays were used for CSF analyses of Aβ42 and p‐tau181.The *CSF measure of abnormal brain Aβ* was the p‐tau181/Aβ42 ratio defined as ≥0.023.^6^

The baseline CSF, amyloid PET, and cognitive function testing came from the same visit. The median (interquartile range [IQR]) time difference between clinical visit and PET was 7 (0, 14) days, and between the clinical visit and CSF was 7 (1, 16) days. There were some longer differences with the maximum difference being 216 days between clinical visit and PET and 217 days between clinical visit and CSF.

### 1.4 Informed consent

This is a secondary analysis of already collected data. The ADNI study was approved by the institutional review boards of all of the participating institutions. Informed written consent was obtained from all participants at each site.

### 1.5 Analyses

We were interested in cognitive outcomes among participants stratified by a four‐level CSF/PET classification (CSF−/PET−, CSF+/PET−, CSF−/PET+, and CSF+/PET+). We compared these groups on their underlying CSF and amyloid PET levels using Kruskal–Wallis and Wilcoxon rank sum tests. The AVLT sum of trials 1–5 exhibits no floor or ceiling effects in MCI participants and is approximately normally distributed. We fit a linear mixed model to estimate mean AVLT sum of trials 1–5 values at baseline and the annual change in AVLT sum of trials 1–5 by group. We included only participants with at least two AVLT tests (i.e., serial data). The model included sex, age, and CSF/PET group as covariates that could affect both initial values and rates of change. Education was included in the model as a baseline covariate, but it was not included in longitudinal models to keep the models tractable given small numbers in the discordant groups. The model further specified that there was heterogeneity across individuals which was not explained by these covariates and which was expressed in terms of between‐participant variation in AVLT values at baseline and between‐participant variation in rate of change. (This heterogeneity was modeled by including correlated random participant‐specific intercepts and slopes.)

The CDRsb was examined for baseline comparisons and changes over time across groups. We fit a longitudinal proportional odds logistic regression model to describe baseline differences across diagnostic groups and differences in the odds of increasing on CDRsb across groups. The model included adjustments for age, sex, and education, allowed rates to vary by group, and included a random subject‐specific intercept. The CDRsb model was more parsimonious than the AVLT model due to the reduced information owing to the more discrete CDRsb. We used a Bayesian approach for model estimation with weakly informative priors to improve estimate stability. Posterior summaries were obtained via Markov chain Monte Carlo with four chains, each with 5000 iterations of burn‐in which we discarded and 5000 iterations which we used for summarizing associations. This model was fit using the **brms** package in *R*.

A third analytical approach was a simple person‐years analysis estimating rates of progression from MCI to dementia using Poisson regression with the outcome coded as 1 if the person obtained a dementia diagnosis by last follow‐up and 0 otherwise. Follow‐up time was included in the model as an offset.

**RESEARCH IN CONTEXT**

1. **Systematic review**: We reviewed published articles that compared longitudinal outcomes in persons with mild cognitive impairment (MCI) who had had both amyloid positron emission tomography (PET) and cerebrospinal fluid (CSF) assays for amyloid‐β42 peptide.
2. **Interpretation**: In contrast to the clinical decline observed in persons who had both elevated brain amyloid and an abnormal p‐tau181/Aβ42 ratio, persons with MCI with in whom the two proxies for brain amyloid were discrepant did not exhibit decline over a follow‐up of 4 years. Thus, the biomarker profile in a person with MCI in which either PET or CSF indicators of elevated brain amyloid are not abnormal is likely indicative of a favorable prognosis.
3. **Future directions**: Similar analyses need to be carried out using plasma biomarkers versus amyloid PET in order to understand better the predictive power of plasma Alzheimer biomarkers for progression of cognitive decline in persons with MCI.

## 2 RESULTS

**FIGURE 1:**
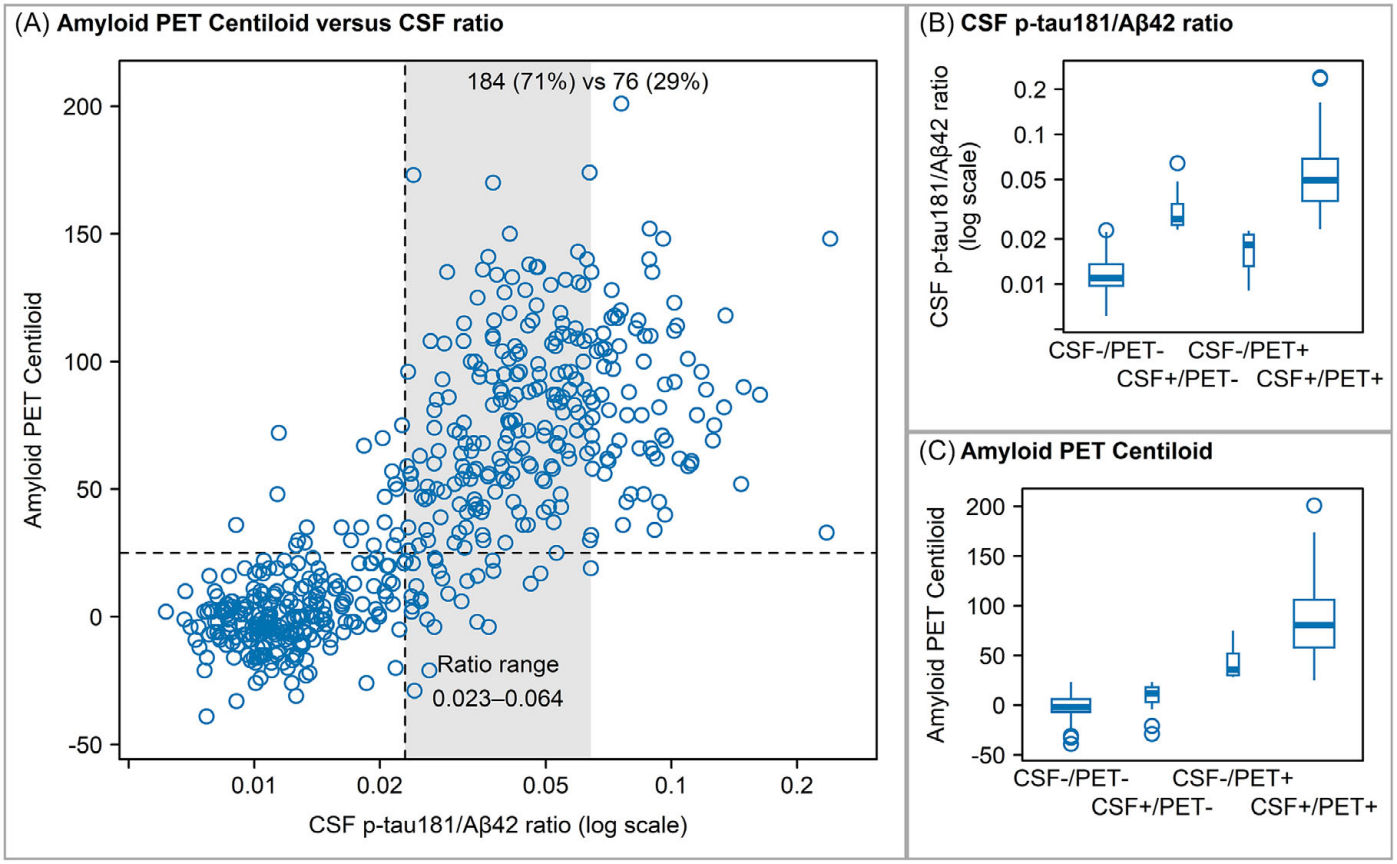
(A) Scatter plot of amyloid PET centiloid values versus CSF p‐tau181/Aβ42 ratio values at baseline. Dashed lines indicate cut‐points of 0.023 for CSF and 25 for amyloid PET Centiloid. The shaded area emphasizes the range of p‐tau181/Aβ42 values in the CSF+/PET− group. (B) Box plots of CSF p‐tau181/Aβ42 ratio values by group. (C) Box plots of amyloid PET Centiloid values plotted by group. Box widths reflect relative sample sizes in each group. CSF, cerebrospinal fluid; PET, positron emission tomography.

The ADNI MCI participants (*n* = 541) had a median age of 72 years, comprised 43% women, 48% apolipoprotein E (*APOE)* ε4 carriers, and had a median duration of follow‐up from first CSF/PET to last clinical visit of 4.0 years (IQR 2.1–6.0 years) (Table 1). There were 511 (94%) who self‐identified as White and 524 (97%) as not Hispanic or Latino. Discordance of Aβ status between CSF and PET was uncommon. For the CSF+/PET− pattern, there were 27/541 (5%) persons, and for the CSF−/PET+ pattern, there were 21/541 (4%) persons (Table 1; see Figure 1A for scatterplot). The CSF−/PET− group was slightly younger than the others, while the sex distribution was roughly comparable across groups, with the exception of the CSF+/PET− group having somewhat fewer women. In comparison to the CSF−/PET− and CSF+/PET+ groups, the CSF+/PET− group fell between the two other groups in proportion of *APOE* ε4 carriers at 48%. The CSF−/PET+ group had a lower proportion of *APOE* ε4 carriers (33%).

**TABLE 1:** Demographics of ADNI MCI participants at their first CSF and amyloid PET.

Parameter	CSF−/PET− (*n* = 233)	CSF+/PET− (*n* = 27)	CSF−/PET+ (*n* = 21)	CSF+/PET+ (*n* = 260)	Total (*n* = 541)
Age, y					
Median (Q1, Q3)	69 (63, 75)	74 (70, 81)	73 (68, 76)	74 (69, 78)	72 (67, 77)
Range	55–92	61–85	60–86	55–89	55–92
Sex					
Female	107 (46%)	9 (33%)	10 (48%)	109 (42%)	235 (43%)
Male	126 (54%)	18 (67%)	11 (52%)	151 (58%)	306 (57%)
Education, y					
12 or less	22 (9%)	4 (15%)	3 (14%)	46 (18%)	75 (14%)
13 to 15	51 (22%)	2 (7%)	3 (14%)	47 (18%)	103 (19%)
16	54 (23%)	6 (22%)	7 (33%)	56 (22%)	123 (23%)
More than 16	106 (45%)	15 (56%)	8 (38%)	111 (43%)	240 (44%)
APOE ε4 genotype					
Non‐carrier	180 (77%)	14 (52%)	14 (67%)	76 (29%)	284 (52%)
Carrier	53 (23%)	13 (48%)	7 (33%)	184 (71%)	257 (48%)
Mini Mental State Exam					
Median (Q1, Q3)	29 (28, 30)	29 (28, 30)	29 (28, 29)	28 (26, 29)	28 (27, 29)
Range	24–30	23–30	23–30	19–30	19–30
AVLT sum of trials 1–5					
Median (Q1, Q3)	40 (32, 48)	39 (30, 43)	38 (31, 46)	32 (26, 38)	35 (29, 44)
Range	18–72	19–56	22–61	12–59	12–72
CDR Sum of Boxes					
Median (Q1, Q3)	1.0 (0.5, 1.5)	1.0 (0.5, 1.5)	1.5 (1.0, 1.5)	1.5 (1.0, 2.5)	1.5 (0.5, 2.0)
Range	0.0–5.5	0.5–2.5	0.5–3.5	0.0–6.0	0.0–6.0
Amyloid PET meta‐ROI, Centiloid (LONI)					
Median (Q1, Q3)	−2 (‐7, 6)	12 (3, 18)	36 (30, 52)	80 (58, 106)	30 (0, 78)
Range	−39–23	−29–23	28–75	25–201	−39–201
CSF Aβ42, pg/mL					
Median (Q1, Q3)	1479 (1093, 2006)	778 (660, 1036)	940 (837, 1097)	670 (524, 814)	888 (654, 1392)
Range	372‐3672	312–1638	586–2198	213–1472	213–3672
CSF p‐tau, pg/mL					
Median (Q1, Q3)	17 (13, 22)	21 (19, 31)	17 (13, 19)	32 (25, 47)	23 (17, 32)
Range	7–52	12–43	10–25	10–97	7–97
CSF p‐tau/Aβ42 ratio					
Median (Q1, Q3)	0.011 (0.010, 0.014)	0.027 (0.025, 0.034)	0.018 (0.013, 0.021)	0.049 (0.036, 0.069)	0.025 (0.012, 0.048)
Range	0.006–0.023	0.023–0.064	0.009–0.023	0.023–0.240	0.006–0.240
Time between CSF draw and Amyloid PET scan, days					
Median (Q1, Q3)	8 (2, 19)	5 (0, 11)	9 (6, 16)	6 (1, 14)	7 (2, 15)
Range	0–154	0–35	0–71	0–119	0–154
Time from first to last visit with CSF and amyloid PET data, y					
Median (Q1, Q3)	0.0 (0.0, 2.1)	0.0 (0.0, 2.1)	0.0 (0.0, 2.0)	0.0 (0.0, 2.1)	0.0 (0.0, 2.1)
Range	0.0–11.2	0.0–8.0	0.0–2.3	0.0–8.4	0.0–11.2
Time from first visit with CSF and amyloid PET data to last clinical visit, y					
Median (Q1, Q3)	4.2 (3.0, 7.9)	3.3 (2.1, 4.6)	4.0 (2.0, 6.6)	3.3 (2.0, 5.0)	4.0 (2.1, 6.0)
Range	0.5–12.2	0.5–11.2	1.0–11.3	0.4–11.9	0.4–12.2

Abbreviations: ADNI, Alzheimer's Disease Neuroimaging Initiative; CSF, cerebrospinal fluid; LONI, Laboratory of Neuro Imagaing; MCI, mild cognitive impairment; PET, positron emission tomography; ROI, region of interest.

### 2.1 Baseline comparisons of persons with MCI

The CSF+/PET− group had a lower p‐tau181/Aβ42 ratio (median 0.027, IQR 0.025–0.034, range 0.023–0.064) compared to the CSF+/PET+ group (median 0.049, IQR 0.036–0.069, range 0.023–0.240) (Table 1, Figure 1B). By way of comparison, 71% of the CSF+/PET+ also had p‐tau181/Aβ42 ratios in the 0.023 to 0.064 range. In the CSF+/PET− group, 7 of 27 had p‐tau181/Aβ42 values that were within 0.002 of the cutpoint. The CSF−/PET+ group had CSF p‐tau181/Aβ42 ratio values that were slightly greater than those of the CSF−/PET− group (Figure 1B); the former had lower Aβ42 levels but similar p‐tau181 concentrations. The CSF−/PET+ group tended to have amyloid PET values in the lower ranges, compared to those of the CSF+/PET+ group: 36 (IQR 30‐52) versus 80 (IQR 58‐106) CL (Figure 1C). Only four of 21 in the CSF–/PET+ group had amyloid PET levels above 60 CL. By comparison, fewer than half (45%, 118/260) of the CSF+/PET+ group fell into that lower range (i.e., 25–75 CL) of amyloid PET.

**FIGURE 2:**
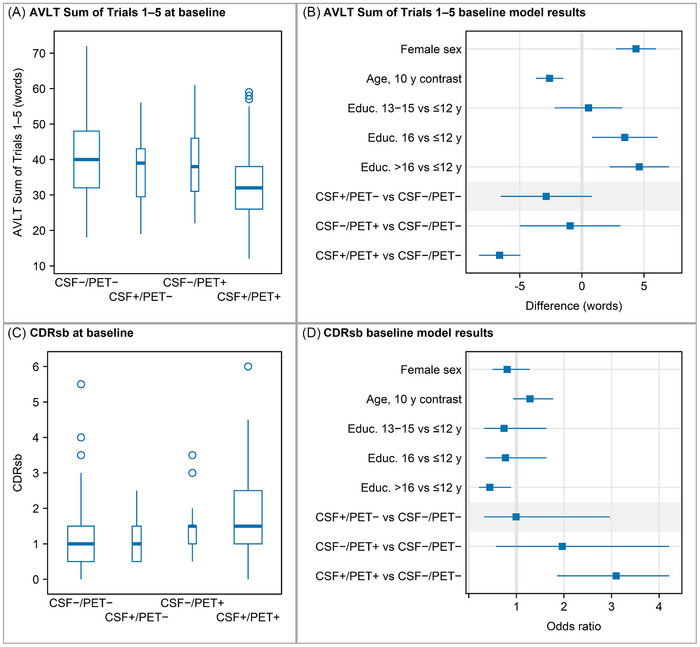
(A) Box plots of AVLT sum of trials 1–5 scores at baseline by group. (B) Associations of sex, age, education, and group with baseline AVLT sum of trials 1–5. (C) Box plots of CDRsb at baseline by group. (D) Associations of sex, age, education, and group with baseline CDRsb from a proportional odds logistic regression model. Box widths reflect relative sample sizes in each group. Model results are summarized with point estimates and 95% CIs. AVLT, Auditory Verbal Learning Test; CI, confidence interval; CDRsb, Clinical Dementia Rating Sum of Boxes.

The CSF+/PET− and CSF−/PET+ groups did not exhibit worse performance on the baseline sum of AVLT trials 1–5, compared to the CSF−/PET− group after adjusting for age, sex, and education (Table 2A, Figures 2A and B). In contrast, the CSF+/PET+ group had lower learning performance compared to the CSF−/PET− group. Both discordant groups also had baseline CDRsb values which were similar to the CSF−/PET− group, while the CSF+/PET+ group had a higher CDRsb consistent with greater cognitive impairment (Table 2B, Figures 2C and D). Pairwise comparisons of the CSF+/PET+ group and CSF−/PET− group were significant (*p* < 0.001); other pairwise comparisons to the CSF−/PET− were not significant.

**TABLE 2A:** Estimates of baseline differences in AVLT Sum of Trials 1–5 by CSF and PET group relative to CSF−/PET− in persons with an MCI diagnosis.

Contrast	Estimate (95% CI)	*p*
CSF+/PET− versus CSF−/PET−	−2.9 (−6.5, 0.8)	0.12
CSF−/PET+ versus CSF−/PET−	−0.9 (−5.0, 3.1)	0.65
CSF+/PET+ versus CSF−/PET−	−6.6 (−8.3, −5.0)	<0.001

Abbreviations: AVLT, Auditory Verbal Learning Test; CI, confidence interval; CSF, cerebrospinal fluid; MCI, mild cognitive impairment; PET, positron emission tomography.

**TABLE 2B:** Estimates of baseline differences in odds of higher versus lower CDRsb by CSF and PET group relative to CSF−/PET− in persons with an MCI diagnosis.

Contrast	Estimate (95% CI)	*p*
CSF+/PET− versus CSF−/PET−	1.0 (0.3, 3.0)	1.00
CSF−/PET+ versus CSF−/PET−	1.9 (0.6, 6.7)	0.29
CSF+/PET+ versus CSF−/PET−	3.1 (1.9, 5.2)	<0.001

Abbreviations: CDRsb, Clinical Dementia Rating Sum of Boxes; CI, confidence interval; CSF, cerebrospinal fluid; MCI, mild cognitive impairment; PET, positron emission tomography.

### 2.2 Longitudinal outcomes in MCI participants

**FIGURE 3:**
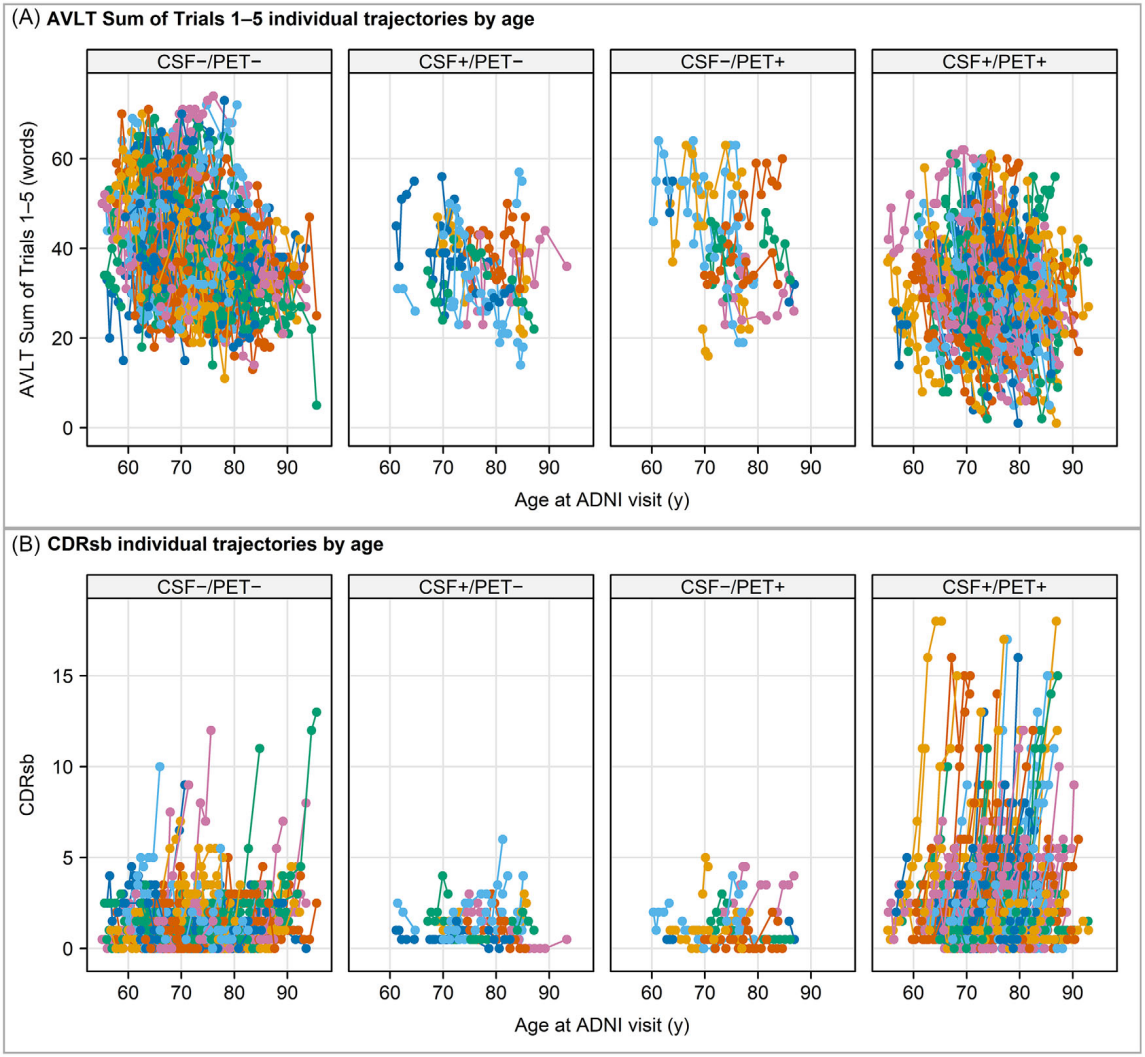
Spaghetti plots of individual longitudinal trajectories in persons with MCI of (A) AVLT sum of trials 1–5, and (B) CDRsb on the *y*‐axis, versus age as the *x*‐axis. AVLT, Auditory Verbal Learning Test; CDRsb, Clinical Dementia Rating Sum of Boxes; MCI, mild cognitive impairment.

**FIGURE 4:**
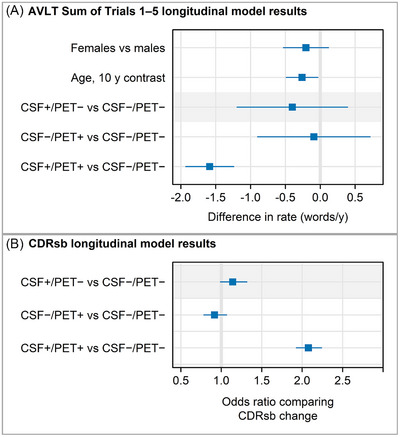
Estimated differences in annual rates of change on (A) AVLT sum of trials 1–5 with 95% CIs and (B) odds ratios with 95% CIs comparing CDRsb change. The AVLT model allowed the rate of change to depend on sex, age, and group. Because of the difficulty of modeling longitudinal changes in CDRsb resulting from the discreteness of the measure, the longitudinal CDRsb model allowed *baseline* CDRsb to vary with sex, age, and education but assumed the rate of change varied only with group. AVLT, Auditory Verbal Learning Test; CI, confidence interval; CDRsb, Clinical Dementia Rating Sum of Boxes.

Spaghetti plots (Figure 3) for the two clinical outcomes depict the individual trajectories of change in each CSF/PET group. The plots for the CDRsb show quite clearly that the two discordant groups were not changing, while the CSF+/PET+ group included many persons who are worsening. Differences in trajectories for the AVLT were difficult to appreciate in Figure 3, but quantitatively, longitudinal change in learning on trials 1–5 of the AVLT showed that after adjusting for age, sex, and education, only the CSF+/PET+ group performed worse than the CSF−/PET− comparison group, by an estimated 1.6 fewer recalled words/year (*p* < 0.001) (Table 2C, Figure 4). Neither discordant group worsened on the CDRsb compared to the CSF−/PET− group. In contrast, the CSF+/PET+ group had twice the odds of worsening on the CDRsb in annual follow‐up compared to the CSF−/PET− group (*p* < 0.001) (Table 2D, Figure 4). Pairwise comparisons of the CSF+/PET+ group and CSF–/PET– group were significant (*p* < 0.001); other pairwise comparisons to the CSF−/PET− group were not significant.

**TABLE 2C:** Estimates of differences in annual rate of AVLT sum of trials 1–5 change (words/y) by CSF and PET group relative to CSF−/PET− in persons with an MCI diagnosis.

Contrast	Estimate (95% CI)	*p*
CSF+/PET− versus CSF−/PET−	−0.4 (−1.2, 0.4)	0.32
CSF−/PET+ versus CSF−/PET−	−0.1 (−0.9, 0.7)	0.82
CSF+/PET+ versus CSF−/PET−	−1.6 (−1.9, −1.2)	<0.001

Abbreviations: AVLT, Auditory Verbal Learning Test; CI, confidence interval; CSF, cerebrospinal fluid; MCI, mild cognitive impairment; PET, positron emission tomography.

**TABLE 2D:** Estimates of differences in time effects of higher versus lower CDRsb at 1 year by CSF and PET group relative to CSF−/PET− in persons with an MCI diagnosis.

Contrast	Estimate (95% CI)	*p*
CSF+/PET− versus CSF−/PET−	1.1 (1.0, 1.3)	0.07
CSF−/PET+ versus CSF−/PET−	0.9 (0.8, 1.1)	0.25
CSF+/PET+ versus CSF−/PET−	2.1 (1.9, 2.2)	<0.001

Abbreviations: CDRsb, Clinical Dementia Rating Sum of Boxes; CI, confidence interval; CSF, cerebrospinal fluid; MCI, mild cognitive impairment; PET, positron emission tomography.

We also examined progression to dementia according to CSF/PET group and found that the rate of incident dementia in the CSF+/PET+ group was 17.3 per 100 persons per year. The rates for the other groups were much lower: 1.9 for CSF–/PET–, 2.7 for CSF+/PET–, and 3.0 for CSF–/PET+ (all *p* < 0.01 compared to the CSF+/PET+ group). There were no significant differences between the discordant groups and the CSF−/PET− group.

Serial PET examinations were available in a limited number of participants in the discordant groups so that no quantitative conclusions could be drawn. However, in the 16 of 27 in the CSF+/PET– group with serial PET, 9 participants showed accumulations of amyloid on PET by last follow‐up but 7 did not. Similarly, in the 13 of 21 in the CSF–/PET+ group with serial PET, 10 showed ongoing accumulation of amyloid by PET. Few serial CSF examinations were available.

## 3 DISCUSSION

There were four major findings from this analysis: First, in MCI patients, the amyloid discordant groups (CSF+/PET– and CSF–/PET+) were small, each roughly in the 5% range. Second, the CSF+/PET‐ group had p‐tau181/Aβ42 values that were not as abnormal as those of the CSF+/PET+ group. Third, the discordant groups had similar baseline memory performance and CDRsb ratings compared to the CSF–/PET– group. Fourth, the discordant groups resembled the CSF–/PET– group in their negligible cognitive decline over a mean of 4 years.

The rarity of discordant CSF and PET in persons with MCI was reassuring for continuing the strategy of using CSF alone to qualify persons with MCI or dementia for AAMA therapy. However, with the proportion of individuals with the CSF+/PET– pattern of about 5%, there would be a 50:50 chance of encountering at least one patient with this discordant pattern over the course of evaluating 14 potential AAMA treatment candidates. In all likelihood, discrepancies between CSF and PET diagnoses of elevated brain amyloid are likely to be greater in clinical practice, where everyday pressures will tend to increase variability in how specimens are handled. Futhermore, quantitation of amyloid PET on the CL scale will vary from institution to institution if it is done at all. However, quantitation has the clear advantage of providing guidance in diagnostic and therapeutic decision‐making that a value is or is not near the diagnostic cutpoint.

The CSF+/PET– group is likely to be encountered in a therapeutic clinic context. In persons with MCI and the CSF+/PET– pattern of results, they were distinguished from the CSF+/PET+ group on the basis of a milder degree of cognitive impairment and lower p‐tau181/Aβ42 ratios. Longitudinal observations in the CSF+/PET– group failed to demonstrate meaningful decline on either a memory test (the AVLT) or worsening on the CDRsb compared to the CSF–/PET– group within the available time of observation. Regardless of the underlying biology, the CSF+/PET– group had a benign prognosis. Justification for AAMA treatment of a person with MCI with the CSF+/PET– pattern would be supported by additional evidence such as an abnormal tau PET scan for likelihood of progression of AD‐related neurodegeneration.

The CSF+/PET– group probably represented several different pathobiological entities. As suggested by subsequent AD biomarker studies, some patients in the group were in the AD pathway. On the other hand, some whose p‐tau181/Aβ42 ratios were close to the cutpoint, might have been persons who truly were not likely to experience AD pathology and therefore not likely to benefit from AAMA therapy. Some of the group could have had a primary tauopathy even if they had AD co‐pathology,^14^ and some might have had an isolated cerebral amyloid angiopathy.^15^ In addition, there were a few MCI patients with very low p‐tau181 and Aβ42 concentrations whose CSF profile could have represented a disorder of CSF dynamics (disproportionately enlarged subarachnoid space hydrocephalus, i.e., the pattern seen in normal pressure hydrocephalus^2^, ^16^). Clinical evaluation and structural imaging would be the key to supporting or refuting the alternative diagnoses.

In the CSF–/PET+ MCI group, the degree of amyloid PET elevation was modest, reflecting either very early Aβ deposition that precedes CSF changes,^17^ or alternatively, a false positive signal due to “bleed‐in” from adjacent white matter tracer retention to cortical voxels. The relative lack of decline in participants with MCI who had the CSF–/PET+ pattern suggested that the isolated, modestly elevated amyloid PET levels in this group represent a more indolent process than that seen in the CSF+/PET+ group.

Because plasma biomarkers are likely to play a larger role in detection of AD biology in the near future, analyses of brain amyloid determinations should be conducted for plasma versus PET concordance in diagnosis and prognosis. From what is currently known for diagnostic applications, the false discovery rates for plasma p‐tau217 (1− positive predictive value) versus amyloid PET are higher than the values for CSF reported here. For example, the positive predictive value for plasma p‐tau217 in several studies were: Wisconsin Registry for Alzheimer's Prevention study, 48% in cognitively unimpaired persons;^18^ Translational Biomarkers in Aging and Dementia study, 82% in a group with a full range of cognitive functioning,^18^ BioFINDER 91% in persons with MCI or dementia,^19^ Swedish Secondary Care 74% in a group with a full range of cognitive functioning,^20^ and AHEAD study 78% in cognitively unimpaired persons.^21^ Longitudinal studies comparing outcomes of plasma biomarkers versus amyloid PET will need to be performed to understand prognosis in persons with p‐tau217 positivity/amyloid PET negativity.

Among limitations of the current report, there are unknown biases in how ADNI recruited participants, possibly raising concerns about the generalizability of the findings from the ADNI cohort. We therefore examined CSF/PET discordance in a Mayo cohort of 621 participants and found discordance to be even rarer. There was only 1 of 90 participants with MCI and 1 of 39 with dementia who had the CSF+/PET– pattern, and 6 participants with MCI and 1 with dementia who had the CSF–/PET+ pattern. No outcome analyses were feasible in the Mayo cohort due to the very small number of discordant cases. The Mayo cohort observations replicated the rarity of discordance in CSF and PET indicators of elevated brain amyloid but could not evaluate the prognosis for discrepant cases.

In summary, persons with MCI from ADNI with the CSF+/PET− or CSF−/PET+ brain amyloid pattern did not exhibit incipient clinical decline, in contrast to persons with the CSF+/PET+ pattern.

## CONFLICT OF INTEREST STATEMENT

David S. Knopman reports no competing interests relevant to this manuscript. He serves on a Data Safety Monitoring Board for the Dominantly Inherited Alzheimer Network Treatment Unit study. He served on a Data Safety Monitoring Board for a tau therapeutic for Biogen (until 2021) but received no personal compensation. He is a site investigator in clinical trials sponsored by Biogen, Lilly Pharmaceuticals, and the University of Southern California. He has served as a consultant for Roche, Samus Therapeutics, Magellan Health, Biovie, and Alzeca Biosciences, but receives no personal compensation. He receives funding from the NIH. Stephen Weigand–no disclosures. Heather Wiste–no disclosures. Jonathan Graff‐Radford receives support from the NIH, serves on the DSMB for StrokeNET, and is an investigator in clinical trials sponsored by Eisai and the Alzheimer's Treatment and Research Institute at USC. Neill R Graff‐Radford is a site investigator in clinical trials sponsored by Biogen, Eisai, Lilly and Cognition therapeutics. Ronald C. Petersen serves as a consultant for Roche, Inc., Eisai, Inc., Genentech, Inc. Eli Lilly, Inc., and Nestle, Inc., served on a DSMB for Genentech, receives royalties from Oxford University Press and UpToDate, and receives NIH funding. Bradley F Boeve received an honorarium for Safety Advisory Board activities for the Tau Consortium funded by the Rainwater Charitable Foundation; he receives grant support from the NIH, the Lewy Body Dementia Association, and American Brain Foundation. Clifford R. Jack Jr. has no financial conflicts to disclose; he receives research support from NIH and the Alexander Family Alzheimer's Disease Research Professorship of the Mayo Clinic. Val J. Lowe serves as a consultant for Bayer Schering Pharma, Piramal Life Sciences, Life Molecular Imaging, Eisai Inc., AVID Radiopharmaceuticals, and Merck Research, and receives research support from GE Healthcare, Siemens Molecular Imaging, AVID Radiopharmaceuticals, and the NIH (NIA, NCI). Mary M Machulda–no disclosures. Julie A Fields reports serving as a consultant to Medtronic. Vijay K. Ramanan has received research funding from the NIH and the Mangurian Foundation for Lewy Body Disease Research; has provided educational content for Medscape, Expert Perspectives in Alzheimer's Disease, and Roche/ADLM; has received speaker and conference session honoraria from the American Academy of Neurology Institute; is co‐PI for a clinical trial supported by the Alzheimer's Association; is site Co‐PI for the Alzheimer's Clinical Trials Consortium; and is a site clinician for clinical trials supported by Eisai, the Alzheimer's Treatment and Research Institute at USC, and Transposon Therapeutics, Inc. Hugo Botha, no disclosures. Stuart J McCarter, no disclosures. David T Jones, no disclosures. Bryan J Neth, no disclosures. Dr. Day reports no competing interests directly relevant to this work. His research is supported by NIH (R01AG089380, U01AG057195, U01NS120901, U19AG032438, P30AG062677). He serves as a consultant for Arialys Therapeutics, and as a Topic Editor (Dementia) for DynaMed (EBSCO). He is a co‐Project PI for a clinical trial in anti‐NMDAR encephalitis, which receives support from NIH/NINDS (U01NS120901) and Amgen Pharmaceuticals. He has developed educational materials for Continuing Education Inc and Ionis Pharmaceuticals. He owns stock in ANI Pharmaceuticals. Dr. Day's institution has received in‐kind contributions for radiotracer precursors for tau‐PET neuroimaging in studies of memory and aging (via Avid Radiopharmaceuticals, a wholly owned subsidiary of Eli Lilly). Kejal Kantarci, no disclosures. Alicia Algeciras‐Schimnich reports serving on advisory boards for Roche Diagnostics, Fujirebio Diagnostics, and received an honorarium from Roche Diagnostics. Joshua Bornhorst reports receiving a speaking honorarium from Roche Diagnostics. Derek Johnson: consultation/advisory board: Novartis (payment to Mayo), Telix (payment to Mayo and personal), Cellectar (personal payment). Alzheimer's Disease Neuroimaging Initiative, not applicable, no disclosures. Author disclosures are available in the supporting information.

## CONSENT STATEMENT

The ADNI study was approved by the institutional review boards of all of the participating institutions. Informed written consent was obtained from all participants at each site.

## Supporting information



Supporting Information
